# Thriving or Surviving? Raising Our Ambition for Trans Children in Primary and Secondary Schools

**DOI:** 10.3389/fsoc.2020.00067

**Published:** 2020-08-11

**Authors:** Cal Horton

**Affiliations:** Education Department, Goldsmiths University, London, United Kingdom

**Keywords:** trans, transgender, child, school, education, youth, primary, secondary

## Abstract

As more trans children find the confidence to make themselves known in our primary and secondary schools, school teachers and administrators look for guidance on how to best support trans pupils. This article synthesises findings from global literature on trans children in primary and secondary education (K1–12 in the US), extracting key themes and conclusions. It then examines the most recent UK school guidance documents on trans inclusion, assessing which lessons and recommendations from global literature are represented. The article highlights existing good practices in visibility and representation and in protection from violence and harassment. Several areas where additional effort is needed are identified, including action on environmental stress and cisnormativity, addressing barriers to school trans-inclusivity and institutional accountability. A number of important shifts are called for: from adaptation on request to pre-emptive change; from accommodation to a rights-based approach; from pathologisation to trans-positivity. Finally, the article raises expectations on what it means to be an ally for trans children in education.

## Introduction

The twenty-first century has seen a global movement away from pathologisation of gender diversity (Bryant, 2006, 2007; Ehrensaft, 2012; Menvielle, 2012; Spack et al., 2012). Gender affirmative approaches to supporting trans children are becoming mainstream (Hidalgo et al., 2013; CASW ACTS, 2015; Ehrensaft, 2016; Murchison et al., 2016; Lopez et al., 2017; Oliphant et al., 2018; Rafferty et al., 2018; Turban and Ehrensaft, 2018). This shift toward gender affirmative approaches is underpinned by evidence of the damage to trans children caused by childhood abuse and rejection, including the harm of conversion therapy (Roberts et al., 2012; Ashley, 2019b; Turban et al., 2019).

There is now strong evidence that socially transitioned children who are supported have good levels of well-being and mental health (Ehrensaft, 2016; Olson, 2016; Durwood et al., 2017; Ehrensaft et al., 2018). Factors associated with improved well-being in trans children include family functioning (Katz-Wise et al., 2018), family support (Travers et al., 2012; Simons et al., 2013; Klein and Golub, 2016), and use of chosen name (Russell et al., 2018). Informed by global healthcare best practices (Oliphant et al., 2018) trans children are increasingly likely to receive love and support in childhood (Ehrensaft, 2016), including transitions at or before primary school (Miller, 2016a; Davy and Cordoba, 2019). Many trans children are aware of their identity at a young age (Jones and Hillier, 2013; Telfer et al., 2015; Fast and Olson, 2018) meaning a majority of educators will at some point work with trans pupils, although teachers may not be aware of the trans children in their classroom (Rands, 2009; Meyer and Leonardi, 2018). Educators can look to a variety of trans inclusion school guidance documents to shape their support for trans pupils of all ages. It is important that these guidance documents are informed by the most up to date literature on trans children. This paper aims to review whether best practices from research literature on trans children in education is feeding through into guidance on supporting trans children in our schools.

Until recently, trans children have been almost invisible in both academic literature and public consciousness, with gender diversity problematised and hidden (Brill and Pepper, 2008; Gill-Peterson, 2018). A majority of older literature on trans children was pathologising and cisnormative^1^ (Ansara and Hegarty, 2012). There has been a recent growth in trans positive research on trans children, looking at the identities of young trans children (Olson et al., 2015; Olson, 2016; Fast and Olson, 2018; Olson and Enright, 2018; Olson and Gülgöz, 2018; Rae et al., 2019; Pullen Sansfaçon et al., 2020), reflecting on social transition (Turban, 2017; Whyatt-Sames, 2017; Turban and Keuroghlian, 2018; Ashley, 2019a,c), and trans children's experiences in education (Bartholomaeus and Riggs, 2017a). Research is now examining the external challenges experienced by trans children and families (Capous-Desyllas and Barron, 2017; Alegría, 2018), including the impact discrimination, and structural cisnormativity^2^ (Hendricks and Testa, 2012; McBride, 2020). This builds upon wider work on the Minority Stress Model (Meyer, 2003); the ways in which experiences of prejudice, stigma and discrimination negatively impact on mental health. Modern, trans-positive research on trans children in education looks at cisnormativity and the structural and systemic barriers to trans children having equality of opportunity in our schools (Bartholomaeus and Riggs, 2017a; McBride, 2020).

In this paper the term “trans children” will be used. Trans children includes transgender and or non-binary children, also described as “gender diverse children” (Keo-Meier and Ehrensaft, 2018). “Cis” is a term for non-trans or cisgender people. Where all ages under 18 are included (including the youngest pre-primary and primary school children) the term children will be used. Where necessary this will be divided either into trans children and trans adolescents and where appropriate the term trans young people or trans youth will be used, with the term youth excluding younger children and including young adults (UNDESA, 2013).

A trans-emancipatory approach informs this research, aiming to proactively avoid pathologisation or cisnormativity, recognising the rights of trans children to equality of opportunity in education. Additional research standards are needed for research on trans communities “because of a long history of intolerance, bias, and psychopathological stereotyping in this specialty” (ITHF, 2019, 1). Vincent (2018) outlines six categories to consider when working on trans-related research including nuanced language use and recognising trans histories. Ethical research needs to consider and address the long and continuing pathologisation of trans children, including past and continuing rights violations, producing research that respects the needs and rights of trans children in education today.

I write this as a parent of a trans child of primary school age and an advocate for trans children's rights. Our trans children are brilliant and spectacular and ordinary—in many ways they are exactly the same as their cis classmates. Yet too often trans children are failed by our cisnormative systems and priorities, too often these children face prejudice and ignorance, too often they shoulder the burden of educating the adults around them. Past generations of trans children have been abused and harmed—lessons need to be learnt today, to build a better world for tomorrow's trans children.

## Research Questions and Methods

This paper comprises two strands, responding to two sequential research questions.

1. What are key themes, findings, and recommendations from research literature on trans children in education?
2. Which of these research driven themes, findings, and recommendations are being prioritised in trans-related school policy and guidance documents?

These research questions are timely for a UK audience, as schools move toward LGBT inclusive primary school Relationships Education (Department of Education, 2019). These research questions are also timely globally, in a period where trans inclusive education is under threat in countries across the world, from anti trans legislation targeting trans children in the US, through to toxic political and media driven debates on trans inclusive education in Australia, Canada and beyond (Bartholomaeus and Riggs, 2017a).

Part one of the research involved an extensive review of global literature on trans children in education. Databases including Educational Research Abstracts Online, Education Research Information Center (ERIC), and Web of Science were searched using paired key words including: transgender^*^/trans^*^ and school^*^, reviewing articles published between 2005 and 2020 inclusive. An in-depth review of article bibliographies supplemented through researcher expertise enabled identification of additional articles (McBride, 2020). Inclusion criteria on literature type were kept broad to include grey literature studies, and to include commentary, review and analysis articles. Only articles published in English were included, and only articles relating to primary and secondary education, with articles on tertiary education excluded. This search identified articles that provide quantitative or qualitative insights on trans pupils' experience in education, looking at literature on trans children under 18, with literature from the last 5 years prioritised. All identified literature was coded using Nvivo software, identifying themes related to trans children's experiences in education. Eight main themes emerged and these are presented in the literature review section.

Part two of the research analysed a selection of guidelines on trans inclusion in education, examining the degree to which themes from the literature review were profiled in the school guidance documents. Relevant primary and or secondary school guidance documents were identified, selecting those which made specific reference to trans inclusion. This included guides on trans children, trans young people, LGBT young people or guides on HBT (Homophobic, Biphobic, Transphobic) bullying. School guidance documents published prior to 2017 were excluded; the most recently released or updated guides were prioritised. Whilst part one took a global overview of literature on trans children in education, part two narrowed the scope to UK school guidance documents—though the findings here are likely to have relevance for educators interested in supporting trans children in any country.

Twelve guidance documents were selected for review, and these are listed in Table 1 below. One guide that is actively transphobic^3^ was excluded. The review did not aim to provide a forensic analysis of any specific guide, but rather to see how well key themes from the literature are represented in school guidance documents. Several of these guides were produced by civil society organisations. Two were from religious organisations (Church of England and Jewish Orthodox). Several were produced by English councils, two were associated with regional devolved government and one was produced by the National Education Union. The 2019 Trans Inclusion Toolkit produced by multiple English Councils is currently facing challenge from groups opposed to trans equality, and later versions of this toolkit currently being revised by local authorities may be significantly different to the 2019 version reviewed here. Notably missing from this list is any England and Wales government endorsed resource, though guidance from the Equalities and Human Right Commission for England Scotland and Wales is anticipated.

**Table 1:** List of trans-inclusion school resources.

Brighton (Brighton and Hove City Council, 2019)	Trans inclusion schools toolkit
CofE (Church of England Education Office, 2019)	Valuing all god's children: guidance for Church of England schools on challenging homophobic, biphobic, and transphobic bullying
EANI (Education Authority Northern Ireland, 2019)	Guidance for schools, EOTAS Centres and youth service on supporting transgender young people
Equaliteach (Equaliteach, 2020)	Free to be: embedding lgbt+ equality and tackling homophobic, biphobic, and transphobic bullying in primary schools
Leeds (Leeds City Council, 2018)	Guidance on supporting children and young people who are trans or who are questioning their gender identity for all schools and children and families services settings
LGBT Youth Scotland (LGBT Youth Scotland, 2017)	Supporting transgender young people: guidance for schools in Scotland
NEU (National Education Union, 2018)	Supporting trans and gender-questioning students
Orthodox Jewish Schools (Chief Rabbi Ephraim Mirvis, 2018)	The wellbeing of LGBT Pupils a guide for orthodox jewish schools
Stonewall BPG (Stonewall, 2018)	Best practice guide: how primary schools are celebrating difference and tackling homophobia, biphobia and transphobia
Stonewall Next Steps (Stonewall, 2020)	Next steps in inclusive education
Stonewall Primary (Stonewall, 2019)	Creating an LGBT-inclusive primary curriculum
Trans Inclusion Toolkit (Trans Inclusion Toolkit, 2019)	Trans inclusion toolkit for schools

## Global Literature Review on Trans Children in Education

This section highlights eight themes derived from a systematic analysis of global literature on trans children's experience in education. The themes include (1) Pathologisation and victim narratives (2) Discrimination and violence (3) Environmental stress and cisnormativity (4) Individual accommodation on request (5) From school panic to affirmation and representation (6) Teacher barriers to action (7) Ambition and allies (8) Child voice and child rights. These themes and findings should shape the ways in which schools support their trans pupils, acknowledging the weaknesses of the current literature including limited consideration of non-binary children, and limited consideration of intersectionality.

### Pathologisation and Victim Narratives

There is a long history of pathologisation, misgendering and invalidation of trans children that cuts across all spheres of society including our schools (Frohard-Dourlent, 2018; Gill-Peterson, 2018). Ansara and Hegarty (2012, 152) highlight the ways in which pathologising or cisnormative language can “dehumanize, silence and erase.” This pathologisation directly affects trans children's experience in school. Riggs and Bartholomaeus (2018) provides an example of a parent of a 5 year old trans girl being asked by a school to provide a psychiatrist report and have genetic testing before the school might accept her. Pathologising approaches can also be expressed in more subtle ways which nevertheless erase and delegitimise (Frohard-Dourlent, 2018), such as when trans children's identities are denied, or replaced with pathologising and delegitimising terms. The avoidance of the word trans when referring to trans children is a form of erasure, that denies trans children “self-intelligibility” (Kennedy, 2018, 135).

There is a tradition of stigmatization and problematisation of trans children (Pyne, 2014; Kennedy, 2018) with trans children defined through their association with trauma (Marx et al., 2017). Educators also need framings that centre joy, romance, laughter, strength, and resilience (Marx et al., 2017; Shelton and Lester, 2018). Trans pupils, living in cisnormative environments, may develop particular strengths, types of cultural capital (Pennell, 2016a). This can include navigational capacity, being able to navigate through systems not designed for trans pupils; linguistic capacity, challenging linguistic norms that marginalise, erase or other trans pupils; familial capacity, finding support from trans peers, trans communities and trans-led narratives; and resistant capital, being able to fight against discrimination and advocate for equality (Pennell, 2016b).

Descriptions of trans children and youth often centre a victim narrative (DePalma and Jennett, 2010), framing them as in need of protection (Marx et al., 2017). This singular and simplistic framing as “at risk” (Frohard-Dourlent, 2018) homogenises, pathologises and others trans youth as inherently separate from healthy cis peers (Miller, 2016c; Marx et al., 2017; Blair and Deckman, 2019). A victim framing also individualises the challenges trans pupils face (Shelton and Lester, 2018), overlooking the structural inequalities harming them (Smith and Payne, 2016).

### Discrimination and Violence

Trans children face multiple areas of overt discrimination, including segregation and denial of access to appropriately gendered spaces (Kennedy, 2013; Kosciw and Pizmony-Levy, 2016; O'Flynn, 2016; UNESCO, 2016; Kuvalanka et al., 2019; Neary, 2019). School-based anti-trans discrimination targeting trans pupils of all ages is apparent in a number of surveys, with trans pupils prevented from using their name or pronoun at school, and forced to use inappropriately gendered facilities (Kosciw et al., 2016, 2018). Harm is compounded when schools enable trans children to be drawn into public debates on whether schools should actively discriminate against trans pupils (Herriot et al., 2018; Miller et al., 2018; Sinclair-Palm and Gilbert, 2018).

Evidence from diverse locations continues to show trans pupils experiencing hostile school climates (Greytak et al., 2009; Grant and Zwier, 2011; Taylor and Peter, 2011; Kosciw et al., 2012, 2016, 2018; Peter et al., 2016; Ullman, 2017; Fayles, 2018; Human Rights Campaign, 2018) with high incidences of verbal harassment, bullying, physical abuse, and sexual harassment (Reed et al., 2010; Human Rights Campaign, 2014, 2018; Kosciw et al., 2016; Peter et al., 2016; Bradlow et al., 2017). Kosciw et al. (2018) found a steady increase in negative remarks about trans people in schools between 2013 and 2017, highlighting that progress is neither linear nor guaranteed. Trans pupils report lack of safety across multiple locations, including in primary schools (Meyer et al., 2016) especially in gendered spaces like changing rooms and bathrooms (Kosciw et al., 2016, 2018). In a 2017 US survey of over 5,000 trans adolescents, only 16% reported that they always feel safe at school (Human Rights Campaign, 2018).

A hostile school climate can have extensive consequences for trans pupils' ability to thrive (Greytak et al., 2009). Trans pupils experiencing harassment and transphobia are less likely to be able to concentrate in class (Robinson et al., 2014), have lower educational aspirations and poorer educational attainment (Greytak et al., 2009; Kosciw et al., 2012; Robinson et al., 2014; Fayles, 2018). Trans pupils report hiding at lunch times, avoiding gendered spaces like bathrooms and changing rooms (Jones and Hillier, 2013; Robinson et al., 2014), and not participating in extra-curricular events and activities due to lack of safety (Jones and Hillier, 2013; Kosciw et al., 2016). Trans youth have high levels of school absenteeism due to harassment (Greytak et al., 2009, 2013; Taylor and Peter, 2011; Kosciw et al., 2012, 2016; Robinson et al., 2014). Lack of affirmative or safe school environments is also associated with trans pupils dropping out of education or transferring schools (McGuire et al., 2010; O'Flynn, 2016).

In-school victimization and harassment are known to have significant psychological and social consequences (Poteat and Espelage, 2007), including negatively affecting psychological well-being (Kosciw et al., 2012), and contributing to high levels of depression, self-harm, and suicidal ideation (Jones and Hillier, 2013). In a 2017 UK survey of around 500 trans pupils aged 11–19, 45% reported that they had attempted to take their own life, and 84% reported self-harm (Bradlow et al., 2017). A negative school climate (combined with wider systemic oppression), leaves trans pupils with low levels of optimism about their chances of future success and happiness (Human Rights Campaign, 2014).

### Environmental Stress and Cisnormativity

Cisnormative school climates place trans pupils under persistent psychological stress (Ullman, 2015b; Miller, 2016c; McBride, 2020). Institutionalised cisnormativity (Bauer et al., 2009) negatively affects trans pupils, delegitimising their identities and making their lives harder in multiple and systemic ways (Miller, 2016c; McBride, 2020). Trans pupils experience persistent microaggressions which they recognize as symptoms of deeply embedded structural inequality and violence (Woolley, 2017), yet schools are likely to view them as individual isolated acts. Schools may already be aware of overt, individualised, intentional acts of transphobia or violence, but they need to also be aware of the compounding effects of subtler acts of cisnormativity, including systemic practices that are not intended to cause harm to trans pupils (Riggs and Bartholomaeus, 2018). Beyond physical safety, trans pupils need to feel emotionally safe and welcome in school (Brill and Pepper, 2008). In the words of one parent of a young primary school aged trans child (Slesaransky-Poe et al., 2013, 30):

“*I needed to know if he would be physically and emotionally safe; feel welcomed, respected, and fully embraced; and be able to focus on learning*.”

A persistently stressful and hostile school climate can make school about survival rather than success and fulfilment (Miller, 2016c), with environmental stressors detrimentally affecting educational achievement and well-being (Ullman, 2017). The educational disadvantage trans youth experience is not individualised, but structural and systemic (McBride, 2020). Trans pupils experiencing macro and micro aggressions (Miller, 2016a) are forced to develop defensive strategies (Greytak et al., 2013; Bowers et al., 2015; Ingrey, 2018; Kennedy, 2018) which are emotionally and cognitively difficult, reducing well-being and the ability to learn and thrive.

Areas of gender segregation can increase the stress felt by trans pupils (Greytak et al., 2013; Kennedy, 2013; Bowers et al., 2015; Ingrey, 2018), placing them under additional surveillance and pressure to conform (Woolley, 2017). Socially transitioned children who have not disclosed their gender modality—that they are trans-carry an additional stress (McGuire et al., 2010) as they navigate systems which assume they are cis^4^.

Approaches that prioritise an individualised anti-bullying discourse, including the UK Government's approach (Carlile, 2019), overlook the systemic nature of the challenge faced by trans children in schools (Ansara and Hegarty, 2012), and distract from the systemic reforms needed to ensure trans children are welcomed as equals at school (Frohard-Dourlent, 2018; Riggs and Bartholomaeus, 2018). We need to move beyond an exclusive focus on safety, on violence and on individual bullies and victims, to understanding and dismantling the systemic operation of cisnormativity in our schools (Payne and Smith, 2014; Miller, 2016c; Frohard-Dourlent, 2018).

### Individual Accommodation on Request

Few schools provide trans-inclusive adaptations prior to having a known trans pupil (Davy and Cordoba, 2019). Many schools only take reactive actions to accommodate a trans pupil on request (Omercajic, 2015; Davy and Cordoba, 2019), often prompted by informed parent advocacy for their trans child (Neary and Cross, 2018; Riggs and Bartholomaeus, 2018; Davy and Cordoba, 2019). Schools often only accommodate access to appropriate bathrooms after pupils or parents request such access (Ingrey, 2018). This approach means trans pupils' access is to be requested, negotiated and permitted. Ingrey (2018) highlights the rights violation of requiring trans pupils to apply for access, rather than the system proactively making trans pupils welcome. Trans pupils are “subjected to an approval process for a simple act of accessing a suitable washroom space; this process is humiliating, pathologising and alienating, and ultimately transphobic” (Ingrey, 2018, 781).

A individualised “accommodation on request” approach leaves the status quo intact, maintaining an “artificial hierarchy” (Serano, 2016, 13) where the dominant gender (Ingrey, 2018) is validated as “natural,” in the process pathologising trans pupils' gender modality (that they are trans—Ashley, 2019d) as requiring approval and exception from the “norm.” Trans pupils' right to identity and basic dignity is dependent on them submitting themselves to a pathologising and daunting process of justifying their needs and their identity to cis teachers or school administrators (Ingrey, 2018). This accommodation may be particularly hard for children who are gender fluid or non-binary (Omercajic and Martino, 2020)—though it needs to be noted that the current literature has little consideration of non-binary children (Airton and Koecher, 2019).

Meyer and Leonardi (2018) conducted interviews with teachers, which found reluctance to make trans-affirmative school changes unless, and until, they personally knew a trans pupil. This is born out in the wider literature, with numerous examples of schools only making changes when forced to do so, when they encountered their first known trans pupils (Slesaransky-Poe et al., 2013; Mitchell et al., 2014; Baldwin, 2015; McBride, 2020). These pioneer children may “shoulder an immense responsibility as singular sites of all learning and change,” becoming “sacrificial lambs” (Meyer et al., 2016, 9), whose privacy and right to equality of education are neglected in order for the school to commence incremental adaptation. Meyer et al. (2016, 9) discuss the “ethical dilemma of this pedagogy of exposure,” and how to prompt trans inclusive school changes without a trans pupil or family being required to expose themselves as educator of an unprepared school.

Supportive parents and carers are relied upon to advocate for their trans children (Neary, 2019), educating their children's teachers, and advising on inclusive policies and curricula (Bartholomaeus and Riggs, 2017a). Families of trans children cannot just presume their children will be safe and welcomed in schools, and instead need to be constantly vigilant, to protect and advocate for their children (Hill and Menvielle, 2009; Johnson et al., 2014; Pullen Sansfaçon et al., 2015; Rahilly, 2015; Riggs and Bartholomaeus, 2018; Neary, 2019). Parental advocacy on behalf of trans children is an ongoing requirement, with support for trans inclusivity not automatically sustained or replicated across a school (Johnson et al., 2014; Riggs and Bartholomaeus, 2018). Effective inclusion needs to be embedded in clear trans-affirmative policies and procedures (Bartholomaeus and Riggs, 2017a), which are developed proactively, rather than enacted upon request (Baldwin, 2015).

An individualised approach—listening to a child's voice, hearing their needs and being guided by a child's own individual path—is absolutely critical to child-centred care (Whyatt-Sames, 2017). Where families are supportive of their trans child, a collaborative trusting relationship between families and schools can help ensure an effective child-focused path to providing a friendly, welcoming school (Slesaransky-Poe et al., 2013). However, this individualised approach should not be a way of shifting responsibility onto pupils (Frohard-Dourlent, 2018) and is not a substitute for proactive structural changes to ensure trans children are made welcome in our schools (Omercajic and Martino, 2020). Frohard-Dourlent (2018) imagines a future where trans pupils don't need to self-advocate, because schools are already set up to recognise their existence.

### From School Panic to Affirmation and Representation

There is a pervasive culture of silence (Ullman, 2014; Ullman and Ferfolja, 2015; Frohard-Dourlent, 2016); around trans lives at school that has a negative impact on trans children (Ryan et al., 2013). This culture of silence is reinforced through multiple means, from formal legislation against LGBT inclusion in school^5^ (Carlile, 2019), to teacher self-censure (Roberts et al., 2007), through to approaches that police offensive language without empowering teachers to provide alternative positive narratives (DePalma and Atkinson, 2009a).

A culture of silence is also promoted by cisnormativity, wherein any trans representation is perceived through a lens of hyper-visibility (DePalma and Atkinson, 2006). Trans (and LGB) equality can be seen as controversial in a way that does not extend to other equalities (Atkinson and DePalma, 2008) with some commentators considering children “too young” to learn about their trans classmates (Bartholomaeus and Riggs, 2017a). The presence and increasing visibility of trans children in primary and nursery classrooms (Riggs and Bartholomaeus, 2018) forces primary school educators to face up to the silence surrounding trans lives (Payne and Smith, 2014). Unprepared schools can enter into “school panic,” when a culture of silence comes up against the reality of trans children's lives (Smith and Payne, 2016; Bartholomaeus and Riggs, 2017a).

“*When marginalised groups begin to challenge society's expectation that they will remain invisible and silent, they are faced with a choice between invisibility (where they have traditionally been assumed not to exist) and surplus visibility (where their mere presence seems excessive)*.” (DePalma and Atkinson, 2009b, 887).

Schools need to adopt a “usualising” approach to trans inclusion, where trans people are destigmatised to the point that their visibility is no longer of note (Iskander and Shabtay, 2018; Carlile, 2019). Trans people can be made part of everyday life through incorporation into different parts of the curriculum (Mitchell et al., 2014) moving trans lives in schools “from surplus visibility to ordinariness” (DePalma and Atkinson, 2009b, 884).

Curricula remain cis-dominated (Miller et al., 2018) with trans identities nearly invisible (Miller, 2016a). Many children do not see any representations of trans people at school (Mitchell et al., 2014; Peter et al., 2016). Erasure of trans visibility delegitimizes trans identities (Miller, 2016c), forming a systemic macro-aggression where trans pupils need to continuously self-advocate and educate to be read as valid (Frohard-Dourlent, 2018). When schools do not affirm or represent trans identities, this impacts on trans children's self-image, belonging and sense of worth (Ullman, 2014; Miller, 2016a). Exclusion from the curriculum gives a message that trans identities are inferior (Miller, 2016c; Shelton, 2016). Marginalisation and exclusion at school and in wider society teaches trans pupils that there is no place for them in the school or the wider world (Ryan et al., 2013; Ullman, 2017).

Affirming trans-positive school environments are vitally important for trans pupils, improving mental health, well-being, self-esteem, school engagement, and sense of belonging (Olson, 2016; Bartholomaeus and Riggs, 2017a; Day et al., 2018). Children who are affirmed at home and at school have positive academic and emotional outcomes (Davy and Cordoba, 2019). Miller (2016a, 6) highlights the importance of schools being affirming with a “pedagogy of recognition” where trans pupils can see that they are valued, not merely tolerated. Trans representation can also have huge importance for gender questioning children, with access to the word trans, and knowledge of the existence of trans identities opening doors to self-discovery (Kennedy, 2018). Most pupils do not see any trans representation in schools (Bradlow et al., 2017), and the representation that does exists is mostly negative, framing trans people as “at risk” (Bittner et al., 2016). In these contexts trans pupils can gain confidence and self-esteem from any positive trans representation (Snapp et al., 2015).

An inclusive curriculum explicitly tackles the misconceptions that underpin transphobia (Meyer et al., 2016) and reinforces peer acceptance (Kosciw et al., 2012; Ryan et al., 2013), with increased peer support creating a more positive school climate for trans pupils (Kosciw et al., 2012; Jones et al., 2016). Trans representation in the classroom sends pupils a message that teachers support them, that they have a right to be safe in school (Kosciw et al., 2012; Peter et al., 2016), that they are not alone (Miller et al., 2018). A trans-affirmative curricula builds a more supportive, welcoming school climate (Peter et al., 2016; Martino and Cumming-Potvin, 2017), and improves well-being of trans pupils (Greytak et al., 2013; Kahn and Lindstrom, 2015).

Trans pupils who feel able to fully participate as an equal in school, being open, when they choose, about their identity and being able to discuss “transitude” (Ashley, 2018, 4) at school, had a greater sense of belonging (Greytak et al., 2009). Trans pupils having a sense of belonging at school correlates to pupil well-being, academic motivation and academic achievement (Kosciw et al., 2012; Ullman, 2015b).

Trans inclusion is needed in education about bodies and puberty (Jones et al., 2016), though with care not to limit inclusion to Relationships and Sex Education, which can be pathologising (Formby, 2015; Carlile, 2019). Trans positive representation in literature provides a “pedagogy of possibility” (Bittner et al., 2016, 2) that disrupts cisnormativity (Cumming-Potvin and Martino, 2018) showing trans people “as part of vibrant, supportive communities, living fulfilling, and productive lives” (Parsons, 2016, 11). Trans representation in history, showing historic fights for rights and visibility, helps validate and give hope to trans pupils, whilst also raising acceptance from cis peers (Snapp et al., 2015).

### Teacher Barriers to Action

Trans pupils can experience bullying, transphobia, ignorance and hostility from teachers and school staff (Reed et al., 2010; Taylor and Peter, 2011; Kuvalanka et al., 2014; Formby, 2015; Bartholomaeus et al., 2017), causing significant harm (Robinson et al., 2014). Teachers can also contribute to a hostile climate through inaction when pupils are facing transphobic harassment (Greytak et al., 2009; McGuire et al., 2010; Robinson et al., 2014; Bowers et al., 2015; Ullman, 2015b, 2017; Kosciw and Pizmony-Levy, 2016). Trans pupils who do not feel supported by their teachers are more than four times as likely to leave school if they encounter discrimination (Jones et al., 2016), with teacher failure to intervene seen as a violation of trust (Meyer and Stader, 2009).

Teachers have enormous power to “affirm or belittle the existence of youth in their classrooms” (Kearns et al., 2017, 12). Some teachers and school administrators are positive, well-informed and affirmative, and even just one supportive and trusted teacher can make a profound impact on a trans pupil's experience of school (McGuire et al., 2010; Mulcahy et al., 2016; Bartholomaeus and Riggs, 2017a; Ullman, 2017; Palkki and Caldwell, 2018). In schools where teachers were protective and affirming (Meyer et al., 2016), consistently intervening to disrupt marginalising behaviour, pupils experienced lower rates of bullying (Greytak et al., 2013; Jones et al., 2016), had lower rates of school absenteeism (Ullman, 2015b; Jones et al., 2016), and higher rates of happiness and self-esteem (Kosciw et al., 2013; Ullman, 2015b). Perceived acceptance from teachers matters as much as protection (Ullman, 2014), with teacher positivity about gender diversity significantly correlated with pupil well-being (Ullman, 2015b, 2017). Trans pupils spoke of the importance of having at least one adult who could advocate for them, help them understand their rights, and help them navigate cisnormative institutional cultures and regimes (McGuire et al., 2010).

A key barrier to trans inclusion is teacher willingness, with some staff not believing it is their job to include or affirm trans youth (Meyer and Leonardi, 2018), or having “barriers to empathy” (Blair and Deckman, 2019, 2). Bowers et al. (2015) notes that school staff will be shaped by negative attitudes, misinformation or transphobia endemic in society. Teachers who were willing to refer to LGB identities in their classroom were less willing to include trans people (Formby, 2015), considering the topic taboo (Pullen Sansfaçon et al., 2015), too complex (Mitchell et al., 2014), or too difficult (Cumming-Potvin and Martino, 2018). However, once teachers tried trans and LGB inclusiveness, they were surprised to find children capable of engaging sensitively and thoughtfully (Carlile, 2019). School staff can be overwhelmed by inertia, aware of the need to support trans pupils, but holding on to pre-established prejudices of trans as an undesirable “deviation” (Frohard-Dourlent, 2018). Teachers and school administrators may wrongly assume the existence of transphobic institutional and legal regulations where discriminatory regulations do not exist (Frohard-Dourlent, 2016).

Teacher lack of knowledge remains a barrier (Pullen Sansfaçon et al., 2015; Bartholomaeus et al., 2017; Carlile, 2019). Ill-informed teachers can do harm, by relying on stereotypes that reinforce prejudice (Mitchell et al., 2014). Teachers may also experience fear and anxiety at the presence of trans children in their classroom (Payne and Smith, 2014; Smith and Payne, 2016; Blair and Deckman, 2019), the arrival of a trans child forcing teachers to become aware of (but not necessarily challenge) cisnormative assumptions and practices. Teachers lacked confidence in how to identify school practices that harm trans pupils, or in how to identify transphobic or cisnormative stereotypes, bias or prejudice in teaching materials (Bartholomaeus and Riggs, 2017a). Teachers preferred to focus on “the problem” of fitting trans pupils into a cisnormative school, prioritising individualised actions like name change, rather than considering wider trans-inclusive adaptations (Smith and Payne, 2016).

Teachers and school staff who have undertaken specific training on working with trans pupils, and those with trans friends or family, had more positive attitudes and greater confidence in working with trans pupils, and were more likely to advocate for their trans pupils (Bowers et al., 2015; Bartholomaeus et al., 2017). Staff who had knowingly taught at least one trans pupil had more positive attitudes on trans inclusion (Bowers et al., 2015; Bartholomaeus et al., 2017), building confidence with experience (Davy and Cordoba, 2019), However, a majority had not knowingly taught a trans pupil (Bowers et al., 2015). Mentorship arrangements between staff with prior experience and staff who are new to this were found helpful in raising confidence, though such support is rare, especially in primary schools (Slesaransky-Poe et al., 2013; Bartholomaeus and Riggs, 2017a; Davy and Cordoba, 2019).

Another barrier was teacher concern about wider community or parental opposition to support for trans pupils. Teachers were likely to assume parents as a whole would disapprove of LGBT inclusion (Depalma and Atkinson, 2010) and used this as justification for not acknowledging gender diversity in their teaching. Without school-set expectations, some teachers were likely to focus on the perceived preferences of trans-antagonistic parents, rather than centring the needs of trans children (Malins, 2016).

Teacher fear, underpinned by the impacts of anti-trans and anti-LGBT legislation, is a significant obstacle (Carlile, 2019), with teachers feeling they needed courage to deliver LGBT inclusive curricula (Atkinson and DePalma, 2008; Carlile, 2019). Teachers avoided the topic (DePalma and Atkinson, 2006; Cumming-Potvin and Martino, 2018), believing they needed explicit permission to talk about it (DePalma and Atkinson, 2009a). Teachers need a network of support to enable and encourage trans inclusivity (Malins, 2016). DePalma and Atkinson (2009a) emphasizes how teachers aiming for LGBT school equality may need extra cross-school support, as they can feel isolated and worry about being perceived as “subversive.”

In locations like the UK, with a history of LGBT-exclusionary school legislation, proactive policy, and school-wide efforts are needed to ensure teachers gain confidence that trans inclusion is not controversial or unusual, but essential and routine (Mitchell et al., 2014). Similar efforts are needed when schools come under the pressure of conservative campaigns against trans-inclusion in education, campaigns that put pressure on schools in many countries to overlook their responsibility toward trans pupils (Jones et al., 2016; Bartholomaeus and Riggs, 2017a).

Reference to legal mandates and government or educational guidance is an important support for teachers and school administrators, making the connection to obligations to provide equality of opportunity safety, and physical and emotional well-being for all children (DePalma and Atkinson, 2009a; Mitchell et al., 2014; Carlile, 2019). Leadership, policies and guidelines from national or sub-national government are particularly helpful to ensuring school commitment (Bartholomaeus and Riggs, 2017b). Governments need to fulfil their responsibilities in providing clear legislation and guidance to uphold the rights of trans children in education (Riley, 2012). Unfortunately, governments are frequently slow on delivering this leadership (Riley, 2012; Neary and Cross, 2018), failing in their duty of care for trans children.

Martino and Cumming-Potvin (2018) reference the ways in which teacher action or inaction in support of trans pupils is influenced as much by media landscape as by formal policy, or the ways equality-related policies are framed and understood through media narratives. In contexts where national policy or media landscape is hostile to trans pupils, schools, and teachers having an ethical commitment to caring for their trans pupils becomes even more important (Miller et al., 2018). Leadership and support at school level are critical for teacher action (Malins, 2016). Class teachers look for assurance that they have their head teacher's backing (Mitchell et al., 2014). In many schools, head teachers (principals) are proactively working to ensure equality of opportunity for trans pupils (Meyer et al., 2016). Equality motivated school board members can play a critical role in ensuring teachers and school leadership have a clear mandate to support their trans pupils, ensuring teachers understand and tackle cisnormativity, providing a welcoming school (Meyer et al., 2016).

### Ambition and Allies

The literature challenges the ambition we should have for trans-inclusivity in our schools, shifting from a focus on protection to school environments that affirm, validate, and welcome trans pupils as equals (Case and Meier, 2014; Snapp et al., 2015; Meyer and Leonardi, 2018; Sinclair-Palm and Gilbert, 2018); The literature emphasises the importance of teacher allies (Meyer et al., 2016) and the need to raise our imagination of what teachers and school administrators are able to do to support their trans pupils (Atkinson and DePalma, 2008; Frohard-Dourlent, 2016). Our ambition for teacher allies needs to move beyond protection of an individual pupil, to being willing to dismantle cisnormative structures, policies, and approaches that delegitimise and marginalise trans pupils (Case and Meier, 2014; Meyer et al., 2016; Peter et al., 2016; Marx et al., 2017; Meyer and Leonardi, 2018).

The systemic inequalities experienced by trans pupils represent a significant human rights issue (Greytak et al., 2009; Ullman, 2014; Martino and Cumming-Potvin, 2016), necessitating a shift from trans inclusion to trans emancipation in our schools (Mayo, 2007). Where there is a systemic injustice, as is the case for trans pupils in schools today, allies have a responsibility to act as advocates for social justice (Gonzalez and McNulty, 2010; Kearns et al., 2017). As well as mentoring and supporting empowerment of individual trans pupils to assert their rights, teacher allies can ensure there is clear communication across the school on trans equality, sponsor LGBT groups, educate school staff, and advocate for pupils' rights and well-being across the school and beyond the school gate (Gonzalez and McNulty, 2010; Case and Meier, 2014).

Teacher education and training needs to move beyond basic education on the existence of trans people and on transphobic bullying (Meyer and Stader, 2009; Parsons, 2016; Bartholomaeus and Riggs, 2017a). Trans pupils wanted teacher training to help ensure staff take action to tackle cisnormativity in educational systems and classrooms, improving equality of opportunity for trans pupils (McGuire et al., 2010; Frohard-Dourlent, 2018). Trans pupils often have a good understanding of the structural factors underpinning the challenges they face in school, and wanted school staff to acknowledge and be proactive in tackling these systemic barriers (McGuire et al., 2010). Training needs to help school staff understand the ways in which school cisnormativity marginalises trans pupils (McGuire et al., 2010), positioning trans pupils as lesser or other (Miller, 2016c; Marx et al., 2017). Trans pupils also wanted school educators to be more active in speaking up for trans rights in external processes and policy fora, helping them overcome areas of structural oppression that impede their access to justice and equality in education and beyond (McGuire et al., 2010).

Bartholomaeus and Riggs (2017a) highlight the many examples of cisgender teachers and school administrators who are already effectively advocating for, and standing up with, trans pupils in schools (Ryan et al., 2013; Martino and Cumming-Potvin, 2017). Feeling safe at school needs to be recognised as the bare minimum to expect for our trans children (Ullman, 2015a) with educators needing to go further and ensure schools are inclusive and affirming (Bartholomaeus and Riggs, 2017a) places where trans pupils belong, where they are loved, where they succeed and thrive (McGuire et al., 2010; Miller et al., 2018). Miller (2016b) aspires to a future where trans pupils are commonplace and normalised, where gender diversity does not lead to macroaggressions or marginalization, where there is trans representation across all school materials and curricula, where schools support and embrace trans pupils of all ages.

### Child Voice and Child Rights

A child-rights informed approach centres trans children's right to an educational experience that is safe, inclusive and affirming, a right to “gender legibility” (Miller, 2016c, 34), in schools where they can have an equitable experience to their cis classmates. Trans children have a right to privacy, to gender marker change, and a right to choose if, and how, and when, to disclose their gender modality (that they are trans) (McGuire et al., 2010). Trans children also have a right to be visible in schools, open about their transitude to their classmates and school. Riggs and Bartholomaeus (2018) provide an example of a parent feeling their child and family had been pushed toward not disclosing their transitude, to simplify the situation for the school and other parents, rather than centring the needs and rights of the child.

There remain incidences where trans children's existing legal rights are not respected and where schools fail in their legal duty toward trans children (Taylor and Peter, 2011; Ingrey, 2018). In countries where trans children have legal protection, schools need to ensure administrators, teachers and pupils are aware of these rights, and mechanisms need to be in place to hold schools accountable when these are not fulfilled (Schindel, 2008). Schools also have a responsibility to advocate for trans children's needs and rights, including through educating unsupportive or unenlightened parents (Grossman et al., 2009). Trans children and supportive parents need to know their rights in order to claim them (Davy and Cordoba, 2019), and in order to challenge where there is ongoing inequality and injustice (Schindel, 2008). Meyer and Keenan (2018) outline the limitations of legally mandated protection of trans children, arguing that beyond an individual trans child's rights, there must be a focus on a school's responsibilities, ensuring there is institutional accountability for systemic change.

LGBTQ clubs (GSA in North America) can provide trans children with peer support in an affirming and safe space, and an escape from ignorance and cisnormativity (McGuire et al., 2010; Taylor and Peter, 2011; Kosciw et al., 2012, 2016; Marx and Kettrey, 2016). Trans youth with access to a GSA report more welcoming school climates, lower rates of victimization, greater feelings of school connectedness, and less school absenteeism (Greytak et al., 2013). GSA members report a greater sense of empowerment (Poteat et al., 2016), can come together to jointly challenge systemic injustice and advocate for changes at school (Greytak et al., 2009; Gonzalez and McNulty, 2010), increasingly prioritising trans related advocacy (Poteat et al., 2018). Trans pupils who feel empowered and know their rights, who framed the discrimination they endure as related to societal and systemic prejudice, were more likely to respond with activism, and more likely to feel optimistic about being able to contribute to social change (Jones and Hillier, 2013; Jones et al., 2016). Luecke (2018) discusses components of a “Gender Facilitative School,” with an emphasis on empowering all children to be advocates and supporters of their gender expansive peers.

Many studies note the resilience of trans pupils, their agency to resist injustices and advocate for themselves and their peers (McBride, 2020). Wernick et al. (2014) emphasise that marginalised youth need to identify and drive their own solutions, including through educating peers to join them in collective action. Kjaran and Jóhannesson (2013) highlight the importance of an emancipatory approach that prioritises listening to trans pupils' stories, including their experiences of encountering and resisting cisnormativity and structural violence. However, minority youth cannot be left to single-handedly challenge ingrained and dominant systems of cisnormativity, and the institutional and systemic discrimination that affects their lives (McBride, 2020).

## Review of UK Trans-Inclusion School Guidance Documents

This section summarises an analysis of a sample of recent UK school guidance documents, examining the degree to which themes, findings and recommendations from global literature are represented. It reviews the eight themes from the global literature, finding varied representation of these themes in UK school guidance documents.

### Varied Commitment to Depathologisation and Trans-positivity

Many of the guidance documents contained instances of pathologisation of trans children. Four types of pathologisation were noted: delegitimisation, problematisation, medicalization, and deficit framing. Guidelines on trans inclusion need a stronger commitment to avoiding pathologisation of trans children within their text, alongside working toward depathologisation of trans identities across our schools.

Across the guides there are a number of examples where language and phrasing others and pathologises trans children. There are examples of misgendering with Education Authority Northern Ireland (2019, 11) describing trans feminine children as “birth assigned males.” Education Authority Northern Ireland (2019) delegitimises younger trans children, referencing an older research study on desistance^6^, that has been heavily criticised by modern research literature as flawed and pathologising (Ehrensaft et al., 2018; Newhook et al., 2018b; Turban and Keuroghlian, 2018). A number of the guides completely avoid the term “trans children” in a way that marginalises and delegitimises younger trans children, for example talking of children who “want to live in a different gender” LGBT Youth Scotland (2017, 10), using the phrase “trans young people” throughout (Equaliteach, 2020) or “children who are questioning their gender identity” (Stonewall, 2018, 12). Each of these phrases has a place, as long as it is alongside and not instead of acknowledgment of the existence of trans children. Other guides including Leeds City Council (2018), Brighton and Hove City Council (2019) and the Trans Inclusion Toolkit (2019), confidently use the term “trans children,” an important step in combatting erasure, stigmatisation and pathologisation. Brighton and Hove City Council (2019) normalises the term trans children across its guide, stating that many trans people are aware of their identities at (or before) primary school. The Trans Inclusion Toolkit (2019) outlines the importance of affirming trans children.

There are a number of examples where guides problematize trans children. Education Authority Northern Ireland (2019, 7) has a section “We do not know exactly why people are transgender,” without asking why people are cisgender, an inherently othering frame. Brighton and Hove City Council (2019, 7) is clear on the need to avoid problematisation of trans pupils: “Avoid seeing the trans or gender-questioning child or young person as a problem and instead see an opportunity to enrich the school community.” There are a number of areas in which trans children are medicalised and pathologised, with Education Authority Northern Ireland (2019, 8,37) making reference to a “clinical condition,” and the suggestion that “medical advice may be sought” when responding to younger children. EANI adopts a pathologising and medicalising framing to social transition, whereas the Trans Inclusion Toolkit (2019) has a depathologised and demedicalised section on social transition, making clear it is not linked to medical services.

Many of the guides frame trans children as inherently at risk and in need of protection, devoting significant space to statistics on bullying, abuse, school drop-out and suicidality amongst trans pupils. Some guides make clear that these negative outcomes are not intrinsic to being trans—Brighton and Hove City Council (2019, 11) states: “many of these problems are not caused by being trans but by society's attitude toward people who are trans.” The Trans Inclusion Toolkit (2019, 12) makes clear that risk factors for mental health are “a result of unsupportive social contexts and responses that they encounter due to prejudice and lack of understanding.” LGBT Youth Scotland (2017) emphasises the pathologisation that underlies much parental rejection of trans children, arguing that schools have an important role to play in depathologising trans identities.

### Strong on Transphobic Bullying—Varied on Protection From Discrimination

Basic guidance on avoiding illegal discrimination is prioritised in many of the school guidance documents, outlining the school's obligations to allow name changes or access to appropriately gendered bathrooms, with many making a connection to relevant legislation including the 2010 Equality Act: “so trans girls because they are girls, can use the girls' toilets” (Trans Inclusion Toolkit, 2019, 25). The guides vary in how clearly they prioritise protecting trans pupils from discrimination, especially in situations where others want to exclude or segregate them. LGBT Youth Scotland (2017, 18) prioritises protecting trans children from discrimination: “If a learner feels uncomfortable sharing facilities with a transgender young person, they can be allowed to use a private facility such as an accessible toilet, or to get changed after the trans young person is done. A transgender young person should not be forced to use alternative facilities simply to make other young people feel more comfortable.” Conversely, Education Authority Northern Ireland (2019, 34) does not fully support trans pupils, suggesting staff need to consider “the needs of other pupils.” Education Authority Northern Ireland (2019) also suggests that a trans pupil's own safety can be a reason to deny them access to their preferred facilities, penalising a pupil for their school's failure to keep them safe. This can be contrasted with guidance from LGBT Youth Scotland (2017, 21) which is clear that “risk assessments should not be used to exclude a transgender young person.”

Many of the guides focus heavily on bullying—in fact several of the sampled guides are specifically on HBT (Homophobic, biphobic, transphobic) bullying. There has been a significant UK investment in work to tackle HBT bullying, including pilot initiatives and an evaluation of the effectiveness of different approaches to HBT bullying (Case and Meier, 2014; Mitchell et al., 2016). There needs to be awareness that HBT guides may not go beyond this topic, into other areas which are critical for trans inclusion and trans equality.

All of the guides discuss transphobic bullying, particularly in terms of intentional transphobia and a focus on a single, peer, bully to victim dynamic. Several guides commit to policing abusive language, though do not prioritise action to address underlying views or education to replace transphobic frames with trans-positive ones. The Trans Inclusion Toolkit (2019) meanwhile emphasizes the need to explore and explain when pupils ask inappropriate questions or use transphobic language. Many of the guides associate transphobia with extreme overtly abusive language. The Trans Inclusion Toolkit (2019) on the other hand centres the voices of trans children, who share their experiences of subtler harassment, providing an annex of trans children sharing examples of inappropriate and invalidating questions.

### Minimal Consideration of Cisnormativity

The school guidance documents have minimal consideration of cisnormativity, or the ways in which persistent cisnormativity manifests as continuous microaggressions, making trans children stressed, unwelcome and emotionally unsafe at school.

A wide number of the guides do not even mention the term cisnormativity (Brighton and Hove City Council, 2019; Trans Inclusion Toolkit, 2019; Stonewall, 2020), and whilst others (Equaliteach, 2020) provide a definition, they do not devote space to unpacking how cisnormativity affects trans children's well-being at school.

Overall, the guides give very limited consideration to the systemic changes which are needed to make trans children welcome in our schools. Most of the guidance documents consider the support needed at the point of a pupil's social transition, but appear unaware of ongoing adaptations that trans pupils may need to tackle institutional cisnormativity (Stonewall, 2020). Brighton and Hove City Council (2019) mentions that trans inclusion requires a whole school and community to shift their thinking and understanding, but does not consider implications in terms of shifts in behaviour or systems. Leeds City Council (2018) mentions the need to build trans youth resilience, without considering school responsibilities to ensure trans youth are not living under stress and discrimination.

Many of the guides, particularly for primary schools (Leeds City Council, 2018), do talk about systemic change when discussing the need to challenge gender stereotypes, including the reduction of gendered restrictions and practices. Although this work is useful to all pupils trans and cis, there needs to be recognition that work on gender stereotypes alone is insufficient to overcome the structural cisnormativity that can make schools a hostile environment for trans children.

### Reactive Rather Than Proactive Approach to Trans Inclusion

Many guides place an important emphasis on listening to each individual child, hearing and understanding their individual needs and preferences. However, most of the guides do not mention the burden an individualised and reactive approach places on the shoulders of trans children. LGBT Youth Scotland (2017) refers to the extent trans pupils have to self-advocate throughout their time in education and the Trans Inclusion Toolkit includes a quote from a trans pupil illustrating the burden when they are forced to educate an unprepared school.

Few of the guides explicitly talk about trans children's rights at school. Stonewall (2020) gives a transition check-list that does not mention explaining to a trans pupil their rights. Education Authority Northern Ireland (2019) particularly fails to centre child rights in suggesting a school's commitment to trans inclusive adaptations will depend on a school's ethos and financial resources. Education Authority Northern Ireland (2019, 19) also fails to centre a child's right to an affirming name or pronoun, framing decisions on pronoun change as a “significant change” to be decided by the school rather than the individual pupil. LGBT Youth Scotland (2017, 28) puts each young trans person at the centre “Ask the young person how they would like you and the school to support them.” Without first explaining to a trans child their rights, they may not be aware of how many accommodations they can ask for, especially given the power dynamics at play between cis adults and trans children.

Many of the guides suggest trans inclusion starts at the point of a school knowingly having a pupil ask about transition. The Trans inclusion toolkit emphasises that schools need to provide a trans inclusive environment regardless of whether they knowingly have trans pupils, considering children who have not yet transitioned, as well as pupils who have already transitioned but not disclosed their gender modality. Many of the guides expect trans pupils to make individualised requests before accessing appropriate facilities and expect trans pupils to submit to a review process before gaining permission (National Education Union, 2018; Brighton and Hove City Council, 2019; Trans Inclusion Toolkit, 2019). The guides do not outline the importance of ensuring trans pupils know their rights, or the importance of proactively communicating trans inclusive policies to the school community. Brighton and Hove City Council (2019, 7) is clear that trans pupils should not be made to feel like they are privileged for getting basic accommodations “No trans pupil or student should be made to feel that they are the ones who are causing problems or that they owe anything to their school in return for changes made to support them.”

Many of the guides talk about individualised discussions for school and curricular adaptation to an individual pupil—for example Brighton and Hove City Council (2019) talks positively about working with a parent to adapt a lesson on reproduction to ensure it was trans inclusive. There is no consideration of how to ensure adaptations are embedded and sustained, no consideration of the pressure this places on individual pupils or families at a time when pupils and families may be unclear on their rights, and no consideration of the inefficiencies of each trans child negotiating individualised solutions rather than learning from existing good practices. The Trans Inclusion Toolkit (2019, 6) makes reference to the burden shouldered by trans pupils tasked with negotiating their own trans-inclusive adaptation—one child expressed hope that the trans inclusion toolkit “will take the responsibility for educating people off me.”

### Moderate Emphasis on Inclusion and Representation

Stonewall (2020) and LGBT Youth Scotland (2017) are strong on arguing that an absence of trans representation in our schools sends a negative message, whereas a trans inclusive curriculum embeds positive messages about trans people into regular teaching, normalising trans people as a usual and valued part of our communities. LGBT Youth Scotland (2017, 32) makes clear the case for representation: “Transgender young people typically wait 4 years before talking to someone about their gender identity. During that time, they may not see or hear anything about transgender people, identities or topics at school.” They also include a quote from a trans pupil “Definitely there needs to be more transgender inclusion in education—we didn't even get a single mention of being trans at my school” (LGBT Youth Scotland, 2017, 32).

The suggested examples of trans inclusion can be limited, with some LGBT guidance documents failing to mention trans examples (Chief Rabbi Ephraim Mirvis, 2018). Stonewall (2019) includes a number of examples, but further consideration could be made on how to ensure teachers conduct discussions in a way that affirms trans pupils. Brighton and Hove City Council (2019) commits to inclusive SRE including in teaching on body parts and puberty—ensuring this is validating both for trans pupils and in the impression conveyed to their cis peers.

There is a suggestion across numerous guides that early years and primary work on gender stereotypes equals support for trans students. All pupils will benefit from work on gender stereotypes, but younger trans pupils, who may or may not be gender non-conforming (Olson and Enright, 2018), need additional areas of action. The Trans Inclusion Toolkit (2019, 17) is very clear on the point at which trans inclusion is appropriate: “The appropriate age to discuss the existence of trans and LGB people is the same time it is appropriate to talk about the existence of heterosexual relationships and the existence of boys and girls.” This can be contrasted to Education Authority Northern Ireland (2019) which talks about age appropriateness, controversy, and recommends inclusion at secondary school—age 11+.

LGBT Youth Scotland (2019) recommends talking about trans professionals in light of their professional achievements and commenting in passing at the end of a lesson that they are trans, normalising trans people and demonstrating inclusion in action.

### Varied Attention to Teacher Barriers

Trans Inclusion Toolkit (2019) mentions that school staff can deliberately misgender trans pupils, and are clear that this is a serious concern, an act of harmful behaviour toward a child; as well as highlighting how teachers can inadvertently cause harm through inappropriate questions. Trans Inclusion Toolkit (2019) explains that poor relationships with teachers can be a risk factor for trans children experiencing poor mental health. LGBT Youth Scotland (2019) provides an example scenario and recommended actions if a teacher is deliberately using the wrong pronoun.

Trans Inclusion Toolkit (2019, 6) also highlights a trans child explaining where teachers can make a difference “If one person in school asks the right questions, uses the right name and the right pronouns it can make such a huge difference to a young person. It can help them carry on and live another day.” LGBT Youth Scotland (2019, 9) “expects all teachers to be positive role models to all young people in showing respect to transgender young people.”

Trans inclusion toolkit ends with an annex of trans child quotes which really highlight the impact when schools fail in their duty of care to trans children, including the ways in which impacts on mental health and self-esteem are undervalued: “*I don't drink all day at school so that I don't have to go to the toilet which means I'm always dehydrated and I get headaches all the time and UTIs. Teachers need to know this because it's easy to ignore all the consequences when it's just our mental health but when it's physical they suddenly listen*.”

Trans Inclusion Toolkit (2019) mentions importance of training to ensure “all teachers feel confident to support trans children.” Brighton and Hove City Council (2019) provides scripts for potential difficult questions from wider parents, but does not examine the prejudices, fears or misconceptions that may impede teachers from being supportive.

Very few of the guides explicitly identify and address barriers that may impede teachers from effectively including and affirming trans pupils. LGBT Youth Scotland (2019) simply states that if staff are reluctant they should be reminded of their duty of care to all learners. The Trans Inclusion Toolkit (2019, 6) alludes to teacher related challenges in a quote from a trans child “This toolkit will help people who are scared and sadly, all too often, unwilling to do the right thing.”

Equaliteach (2020) dedicates space to identifying and addressing barriers to teachers creating affirming schools, including ensuring support for younger trans children. Equaliteach (2020) considers mandate, leadership, governance, staff support, working groups and efforts to institutionalise and sustain change.

### Low Expectations for Allies and Outcomes

The documents presented a fairly low bar on teacher and staff expectations, with ambitions framed primarily in terms of being a supportive listener and intervening in transphobic bullying. There was little discussion of practical steps teacher allies can take to disrupt cisnormativity, to reduce the stress carried by trans pupils navigating a cisnormative world.

Equaliteach (2020) framed teacher allies as people who would challenge a HBT incident, who are aware of their legal duties and who understand basic trans related terminology. The level of ambition in LGBT Youth Scotland (2017) in terms of standing up for trans children in primary schools is also very low, prioritising avoidance of gender stereotypes. Stonewall (2020) is a document on next steps in LGBT inclusion, but it is still very basic in its ambition on trans affirmative education. It largely focuses on gender stereotypes, on the fact trans people exist and on supporting pupils who transition to access their legal minimum rights, without commitment to more systemic change.

Brighton and Hove City Council (2019, 6) sets its ambition higher on “developing whole school policy and practice that will allow trans children and young people to achieve at school.” However, the included parent case studies imply satisfaction at basic accommodations of trans children in schools, giving an impression that a school not discriminating against trans pupils is a success. One case study written in a partially positive way included examples of significant school failure—a child never going to the toilet at school—suggesting low parental expectations.

Trans Inclusion Toolkit (2019, 13) calls for higher ambition, emphasising that poor outcomes are not intrinsic to being trans and that schools have a crucial responsibility: “It is vital that schools and education settings don't present negative outcomes as expected futures to children and families. Staff should work to mitigate risk, safeguard children and families and support the development of positive outcomes.”

### Varied Commitment to Child Voice and Child Rights

The school guidance documents vary in how effectively they centre trans children's rights, and in how much they listen to trans children's voices, experience, concerns, and priorities.

The guides vary in their commitment to child rights. Several guides reference the UN Convention on the Rights of the Child (Trans Inclusion Toolkit, 2019), outlining that trans pupils “should be involved in all decisions affecting them, understand any action which is taken and why; and be at the centre of any decision making” (LGBT Youth Scotland, 2017, 8). Several guides reference trans children's right to privacy (Brighton and Hove City Council, 2019; Trans Inclusion Toolkit, 2019), and their right to an education free from discrimination and prejudice (LGBT Youth Scotland, 2017). Brighton and Hove City Council (2019) states categorically that trans children have the right to access correct facilities, though in one section appears to qualify this right: “As far as possible, trans pupils and students should be able to sleep in dorms appropriate to their gender identity.”

The degree to which guides centre child rights is central to their approach to social transition. Education Authority Northern Ireland (2019, 19) describes their approach as informed by a child rights based approach, yet balances a trans child's wish to transition with “the need to protect the child from the negative reaction of others.” Decision making around transition is thus put in the hands of adult staff—likely to be cis. A genuinely child-centred approach is found in LGBT Youth Scotland (2017), which is clear that decision making on transition timelines is in the hands of the trans pupil, stating that “delaying a transgender young person's wish to transition could negatively affect their mental health.” Brighton and Hove City Council (2019) and the Trans Inclusion Toolkit (2019) both emphasise that a school's duty when working with parents or guardians is to represent the interests of the child, to ensure the child's voice is heard and to be their advocate.

The guides vary in how much they centre trans children's voices, with the Trans Inclusion Toolkit (2019) strong on including trans children's quotes throughout. Brighton and Hove City Council (2019) is a third edition that has been developed and informed by “the voice of trans children, young people, adults and their parents and carers.” In terms of giving platforms for trans children to share their experiences, Stonewall (2020) includes an example of a secondary school where confident trans pupils are invited to give assemblies to all students on their experiences and how others can help. However, the example from a primary school is just on gender stereotypes, leading to a question on whether there is prioritisation on hearing the voice of younger trans pupils.

Many of the guides explicitly prioritise asking pupils prior to a social transition their views on their needs and preferences in terms of their individual transition. There is not a similar commitment to asking trans pupils who have already transitioned to share their views on systemic changes that would make the school better for themselves and current and future trans pupils. Education Authority Northern Ireland (2019) however, did include examples of gaining the views of trans pupils through meetings with the deputy head and through a confidential survey.

The Trans Inclusion Toolkit (2019) has a trans inclusion checklist for a school to self-complete, likely from the perspective of cis teachers or school administrators who do not directly experience the challenges that trans pupils face in a cisnormative school. It would be more ambitious if schools sought a trans pupil perspective on whether the school is matching up to its ambitions on trans inclusion.

## Conclusion

In terms of trans-positivity, many, but not all, of the recent school guidance documents are prioritising affirmative language when writing about trans children. Language plays an important role in how school staff understand and engage with trans children and adolescents, and efforts to avoid pathologisation and ensure trans-positivity are critical. The majority of documents included emphasis on trans visibility and representation, though only few of them outline the reasons for the importance of visibility. Some schools provide visibility targeted at trans pupils, so they can see a positive future for themselves, while failing to note the equal importance of trans visibility for cis pupils, to increase the legitimacy of trans lives, and to reduce the burden on trans pupils to educate and explain themselves to their peers. For trans pupils in our nursery, primary and secondary schools, questioning and ignorance exert a daily toll, a burden that is unreasonable for young shoulders to carry. Visibility and representation have multiple important benefits, letting trans pupils know that they are not alone, giving reassurance that the school supports and will stand up for trans pupils, providing a sense of school belonging to trans pupils, and legitimising trans pupils in front of their cis peers.

The literature identifies some shifts needed in trans inclusive education. At the heart this represents a shift in expectations and ambition for trans pupils, from aspiring for resilience and protection from violence and abuse, to aspiring for self-confident, secure pupils who are validated and represented both in daily school life and across the curriculum, children with equality of opportunity to their cis peers, pupils who can excel and thrive at school. In one interview a school head teacher approvingly remarked that trans pupils weren't looking for basic safety or basic access to facilities, they “wanted everything to change” (Sadowski and Jennings, 2016, 83). Given the growing numbers of trans children confident to come out in our schools (Telfer et al., 2015), schools need to give greater consideration of how they can ensure the well-being of these children, helping them grow up as happy, healthy and supported members of our schools (Neary, 2019).

One critical shift is from a narrow definition of school safety to a focus on emotional safety. Violence and transphobic abuse remain serious concerns for too many trans pupils. Yet, even in contexts where schools have a commitment to protection from transphobic bullying, trans pupils experience cisnormative microaggressions that impact on their well-being. Commitment to tackling intentional transphobic bullying is very important, but it is only a first step toward a positive school climate for trans pupils, not an end goal. Underpinning this shift is commitment to understanding the ways in which trans pupils experience and are negatively impacted by systemic cisnormativity, the additional burden this places on trans pupils' shoulders and the cumulative toll it takes. Cisnormativity contributes to minority stress (Meyer, 2003), negatively impacting on well-being and mental health.

A second key shift is in acknowledging the barriers to trans-emancipatory education. There are pressures and disincentives to trans-inclusive practice that need to be recognised and strategically addressed. These barriers include the culture of silence surrounding LGBT and especially trans lives in schools, with educators in the UK still constrained by the after-effects of discriminatory legislation such as section 28. Teacher attitudes and confidence combine to create a second barrier that needs to be addressed, with a complex interplay between teachers who are prejudiced, teachers who feel creating a trans inclusive atmosphere is political (in a way that cisnormativity is not considered political), teachers who deprioritize the needs of trans children in view of assumed parental conservatism, and the teachers who feel under-trained or under-supported to act. Clear leadership is essential, and in the absence of strong trans-inclusive leadership at national level this leadership and commitment needs to be shown by governors, head teachers and individual members of staff.

A third shift that is needed is from individualised accommodation to proactive adaptation. Many school staff undergoing trans-inclusivity training perceive it as not directly relevant to their practice as they do not knowingly have a trans pupil in their class. However, given prevalence estimates, most teachers will have taught a trans pupil, they may just not have been aware. At present, trans-inclusive adaptations are too often prompted by a specific pupil, a “sacrificial lamb” who sends a school into “panic,” and for whom individualised adaptations are made, adaptations that may not be sustained or transferred to wider classes. The pressure that this individualised approach puts on pioneer children and families is immense and unreasonable. The current emphasis on following an individual child's needs and preferences is absolutely critical, but this should not be a replacement for schools making pre-emptive and sustained changes to benefit current and future trans pupils.

The fourth shift that is required is from accommodation to a rights and responsibilities based approach. Current emphasis in schools is on asking trans students what they want and seeing what adaptations can be accommodated, a “negotiation” approach. Trans pupils and families may not be aware of their rights, or may be uncomfortable claiming their rights. A child rights based approach emphasizes the entitlements that trans pupils have, and is clear that these rights are neither negotiable or limitable. There also needs to be a shift from individual rights to institutional responsibilities, ensuring schools are fulfilling their duty of care to trans pupils.

Lastly, we must raise our ambition of what it means to be an effective ally to trans pupils. The bar needs to be raised from a basic level of respect—using correct pronouns, not discriminating against trans pupils, intervening in cases of abuse—to shifting the systemic injustices that harm trans pupils. Integral to this is an understanding of cisnormativity within education systems and cultures, and the ways in which cisnormativity privileges cisgender individuals and makes life harder for trans pupils. Trans pupils shoulder a triple burden of persistent often unintentional delegitimisation; having to, often single-handedly, educate about gender diversity and cisnormativity; and having to self-advocate for their right to a trans-inclusive school. In the absence of effective and informed allies, trans pupils face this challenge alone. This burden is even harder to bear for the many trans pupils facing additional stresses, including those who have unsupportive or abusive families, those facing harassment and hate inside and outside of school, and those with wider intersecting axes of marginalisation including disabled trans children, neurodiverse trans children and trans children of colour.

## Limitations and Areas for Further Study

This study has relied on secondary data, including analysis of existing literature and school guidance documents. The literature review provides limited insight on intersectional experiences, for example on the experiences of disabled, neurodiverse or black trans children. The literature has limited consideration of non-binary or gender fluid children, and limited data on trans children who are not supported at home. Finally the literature has limited first-hand accounts from trans children of their experiences, especially from younger pre-adolescent trans children. The conclusions and recommendations in this study can be strengthened through interviews and analysis with key informants including teachers, youth workers and parents of trans children. The research can be further improved by targeted interviews with trans children, gaining their insights into the findings and recommendations outlined above, additional steps that will be part of ongoing PhD research by the author.

## Author Contributions

The author confirms being the sole contributor of this work and has approved it for publication.

## Conflict of Interest

The author declares that the research was conducted in the absence of any commercial or financial relationships that could be construed as a potential conflict of interest.

## Footnotes

^1^Cisnormativity is the assumption that everyone is cisgender or should be (Keo-Meier and Ehrensaft, 2018). Serano talked about the concept as a societal double-standard that conveys social and legal legitimacy on cis people's identities, with cis identities “taken for granted and considered valid in a way that trans people's are not'', providing cis people with an advantage (Serano, 2011, 29).

^2^Structural cisnormativity exacts disproportionate harms on trans people, threatening their health, reducing their security and limiting their opportunities (Newbury, 2013). Daily, unpredictable, distressing encounters with structural cisnormativity, leaves trans people in a “constant state of alert” that “manifests as a persistent level of stress unknown to their cis counterpart” (Newbury, 2013, 2).

^3^See https://www.stonewall.org.uk/node/62946

^4^Cisitude—the state of being cis—the antonym of transitude (Ashley, 2018)

^5^Section 28 was a statute in place in the UK between 1988 and 2003, that banned the “promotion” of same-sex relationships in schools (Carlile, 2019).

^6^Itself a problematic and pathologising concept (Newhook et al., 2018a).
